# Prenatal genetic testing and potential consequences

**DOI:** 10.1515/medgen-2026-2008

**Published:** 2026-04-16

**Authors:** Sarah F. Jauch, Philipp Klaritsch

**Affiliations:** Medical University Graz Department of Obstetrics and Gynaecology, Research Unit for Foetal Medicine Auenbruggerplatz 14 8036 Graz Austria; Medical University Graz Department of Obstetrics and Gynaecology, Research Unit for Foetal Medicine Auenbruggerplatz 14 8036 Graz Austria

**Keywords:** prenatal genetic testing, invasive diagnostics, amniocentesis, prenatal drug therapy, intrauterine therapies

## Abstract

Prenatal testing for genetic disorders is part of routine clinical practice. Trisomy 21 can now be detected with high sensitivity using non-invasive tests on maternal blood. In cases with other suspected genetic anomalies or structural malformations, invasive testing is required and can be performed with low risk of complications. Genetic analyses such as microarray and trio whole exome sequencing have led to improved diagnostic yield but can also cause uncertainty and difficult counselling situations. Prenatal testing for genetic disorders in the foetus raises ethical and legal questions: Some couples may wish to exercise their ‘right not to know’, while others might take legal action if a genetic abnormality that could have been diagnosed prenatally and would have justified termination of the pregnancy for the couple is only detected after birth. More recent approaches explore prenatal drug therapies based on specific disease-causing variants.

## Introduction

In 1956, Fritz Fuchs and Povl Riis [1] reported one of the first amniocenteses to determine foetal sex by analysing Barr bodies [2]. That was even before the first sonographic images of a foetus in utero were published in the late 1950s [3, 4]. Eleven years later, karyotyping from cultured amniotic fluid cells was described [2, 5]. Advances in foetal sonography not only enabled increasingly detailed assessment of foetal anatomy, but also precise, real-time ultrasound-guided puncture techniques for amniocentesis (AC) and later for chorionic villus sampling (CVS), which were published in the 1970s and 1980s. [4, 6, 7]. Another milestone in prenatal screening for genetic disorders was the detection of cell-free foetal DNA in maternal blood, which subsequently paved the way for the establishment of non-invasive prenatal testing (NIPT) for common foetal aneuploidies [8, 9]. Meanwhile, prenatal screening for malformations and common autosomal aneuploidies, as well as invasive genetic diagnostics, has become an integral part of routine clinical practice. In recent years, the first targeted prenatal treatments for specific genetic diseases have been developed.

## Prenatal screening tests for genetic aberrations

In the late 1990s, first-trimester screening at 11+0 – 13+6 weeks of gestation (corresponding to a crown-rump length of 45–84 mm) was introduced to test for common foetal aneuploidies. By a combination of nuchal translucency, foetal heart rate, maternal age and maternal serum biochemistry (free beta-human chorionic gonadotropin (beta-hCG) and pregnancy-associated plasma protein-A (PAPP-A)) an individual probability for foetal trisomy 13, 18 and 21 can be calculated. With this method a detection rate of about 90 %, 97 % and 92 % for trisomies 21, 18 and 13 can be reached at a risk cut-off of 1:100 and a false-positive rate of 4 % [10]. In cases with high risk (> 1:50) invasive diagnostic procedures are recommended, while in the intermediate risk group (commonly defined between 1:50 and 1:1000), additional sonographic markers (nasal bone, tricuspid valve and ductus venosus flow) or NIPT should be offered for further risk stratification, whereby NIPT has a higher detection rate (about 99 % for trisomy 21) and lower false positive rate (about 0,1 % for trisomy 21) [11]. The German Society for Ultrasound in Medicine established ‘ten golden rules’ for NIPT [12]: The test should only be performed in combination with an ultrasound scan by a certified examiner to rule out other malformations which would require invasive testing. It is recommended to exclusively test for the three most common autosomal aneuploidies, namely trisomy 21, 18 and 13. The NIPT result still represents a probability, not a diagnosis. Abnormal findings are usually clarified by amniocentesis, as CVS cannot rule out placental mosaicism. Prenatal screening tests that can provide information about underlying genetic disorders also include sonographic screening for foetal malformations in the second and third trimester. This is particularly the case when several foetal abnormalities are combined or when there are malformations that carry an increased risk of certain genetic aberrations [13] like duodenal atresia or atrioventricular septal defect for chromosomal abnormalities such as trisomy 21 [14, 15, 16].

## Common indications for prenatal invasive genetic testing

**Figure 1:**
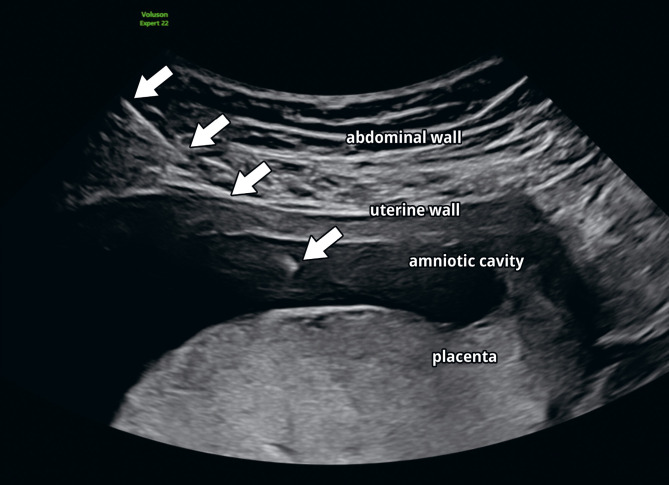
Amniocentesis at 16+4 weeks of pregnancy in a patient with a high risk for trisomy 21 in the combined first-trimester screening due to suspicious maternal serum biochemistry values. From top/outside to bottom/inside, the following structures can be seen: the abdominal wall, the uterine wall, the amniotic cavity, and the posterior placenta. The white arrows indicate the puncture needle with its tip in the amniotic cavity.

Prenatal invasive genetic testing is usually performed by CVS after 10+0 weeks of pregnancy or by AC starting from 15+0 weeks of pregnancy. Figure 1 demonstrates the puncture of amniotic fluid. Typical indications (Table 1) include, for example, an increased risk of a chromosomal disorder in the combined first-trimester screening test or NIPT [13]. According to AWMF guidelines, prenatal invasive genetic testing should be offered if the nuchal translucency measurement is 3.0 mm or higher [11]. If the karyotype is unremarkable, a molecular genetic test like microarray/trio exome sequencing should be provided, especially if nuchal translucency exceeds 3.5 mm [11] or structural anomalies are detected. Similarly, molecular genetic analysis should be recommended for pregnancies with PAPP-A and/or free beta-hCG values <0.2 MoM or beta-hCG values >5.0 MoM [11]. NIPT test failure carries an increased risk of aneuploidy and should be further investigated [17–19]. In addition to foetal malformations, severe foetal growth restriction (FGR) may also be an indication for invasive testing [20], preferably in cases with severe early-onset FGR in the absence of other signs of placental insufficiency. Other reasons are known familial genetic predispositions and significant maternal concern about genetic diseases [13]. If a puncture is performed for another medical reason, such as an infection during pregnancy, cordocentesis for transfusion due to foetal anaemia, amniodrainage for polyhydramnios, or an intrauterine procedure for monochorionic twins, routine genetic testing may also be considered.

Additional molecular genetic testing – microarray/trio whole exome sequencing – may also be indicated in certain foetal abnormalities with unremarkable cytogenetics. Studies have shown that this is important for diagnosis in an additional third of cases [21–23]. Furthermore, special tests are necessary to detect certain genetic diseases, such as methylation analyses for Beckwith-Wiedemann syndrome. However, prenatal methylation analyses are limited, especially in the early weeks of pregnancy, as the methylation process might not be completed [24, 25].

**Table 1:** Common indications for prenatal invasive testing

Increased risk after first trimester combined screening: >1:50 or even >1:100
Increased nuchal translucency: ≥3 mm, but no later than >3.5 mm
Abnormal biochemical findings: PAPP-A <0.2 MoM or beta-hCG <0.2 or >5 MoM
Abnormal NIPT screening results
Repeated NIPT test failures
Foetal malformations
Foetal growth restriction, especially if severe/early-onset
Increased maternal age >45 years
History with increased risk for genetic anomalies
Request of the pregnant woman due to concerns about genetic abnormalities

## Potential consequences/value of prenatal genetic testing

### Risks of prenatal genetic testing

The integration of screening for certain chromosomal disorders into routine clinical practice implicates not only the risk of selection but also the risk that pregnant women feel pressured or undergo testing without having been sufficiently informed about its possible significance and consequences. There is also a risk of stigmatisation and discrimination against affected individuals and couples raising a child with special needs [26]. In accordance with the Austrian Act of Genetic Engineering (‘Gentechnikgesetz’) § 69, it is mandatory to provide non-directive information about the possible scope and significance as well as about possible medical, social and psychological consequences before any examination. The ‘right not to know’ is also enshrined in the Austrian Act of Genetic Engineering. Limitations, technical aspects and risks need to be addressed [13]. Before any genetic examination, written consent should be obtained from the patient after detailed informing and counselling. On the other hand, examiners are under considerable medico-legal pressure, as serious genetic problems that remain undetected during prenatal testing often may lead to lawsuits for compensation. Therefore, it is mandatory that invasive genetic testing is offered to all women with suspicious prenatal findings.

In contrast to earlier assumptions, the procedure-related risk of invasive diagnostic procedures is nowadays considered to be low. A large meta-analysis – considering pregnant women with comparable risk for foetal chromosomal abnormalities in the intervention and control group – showed a procedure-related miscarriage risk following AC of only 0.12 % [95 % CI: –0.05 to 0.30 %; I^2^=44.1 %] and following CVS of 0.11 % [95 % CI: –0.29 to 0.08 %; I^2^=0 %] [27]. Amniotic fluid leakage after AC does not always result in miscarriage, as spontaneous sealing of the membranes is common [13, 28]. Rare complications described include foetal or maternal injuries or organ damage, infection and even sepsis. After CVS, especially after a transvaginal approach, vaginal bleeding occurs more frequently. After any intrauterine procedure, the risk of Rhesus D sensitisation must be considered, and prophylactic anti-D immunoglobulin must be administered to non-sensitised Rhesus-negative pregnant women within 72 h post-procedure unless the foetus is definitely Rhesus negative [13]. Sample mix-ups, maternal contamination, technical non-evaluability, false negative or positive results, genetic disorders that are not yet detectable, and placental mosaicism are other possible risks.

More detailed genetic analyses may also reveal additional findings regarding the parents, such as an increased risk of cancer. Genetic test results can also lead to relationship conflicts or have an impact on other family members [29], for example, if there is a recurrence risk or if it is proven that the partner cannot be the biological father of the child. This should be addressed before testing [29]. The handling of variants of uncertain significance (VUS) should also be discussed. VUS can be classified into different categories. Most genetic laboratories do not report VUS unless they are highly suspicious (‘hot’) VUS. Reporting also depends on the association with the foetal phenotype, the severity of the potential disorder, the time of onset and possible treatment options. An undifferentiated report of VUS carries the ‘burden of uncertainty’ regarding their clinical relevance, which can be stressful and problematic for expectant parents [30, 31]. Additionally, ethical questions arise because the foetus cannot make use of its ‘right not to know’ [32]. This contrasts with the reproductive autonomy of the expectant parents [29]. There is also ongoing debate about who should defray the costs of genetic examinations.

### Prenatal care and mode of delivery

Depending on specific genetic findings, counselling can be difficult, considering variants with variable penetrance and prognosis. Pregnant women with foetal genetic abnormalities usually require interdisciplinary counselling and medical attendance [29]. Intensive foetal care is often indicated, for example, regarding foetal growth development or the occurrence of associated malformations. Maternal care concerning possible associated risks such as mirror syndrome in patients with foetal hydrops is also important [33]. The place of birth should be chosen according to the expertise and necessary neonatal specialist disciplines available. Genetic disorders may also have an impact on the mode of delivery. For instance, in cases of known haemophilia, intrapartum invasive procedures like foetal scalp electrodes and vacuum extraction should be avoided [34, 35], which in some cases may lead to a more generous indication for secondary caesarean section in the event of intrapartum non-reassuring foetal heart rate tracings. Possible consequences for subsequent pregnancies should also be considered. Furthermore, parents whose foetus is diagnosed with a malformation or genetic aberration or who are considering terminating the pregnancy might benefit from psychological support, although the evidence for specific interventions is limited [36].

### Termination of pregnancy

Any abnormal prenatal finding should be communicated to expectant parents in a comprehensible manner. Sociocultural and religious backgrounds must be respected in this regard. Consultations with neonatologists, relevant paediatric specialists and geneticists can contribute to a better understanding, essential for informed decision-making. Contact with other affected individuals or support groups, as well as professional psychological support, might be beneficial. This also includes possible support options which could be helpful when continuing the pregnancy.

Studies from the United States and Japan demonstrate termination rates of approximately 80–100 % for prenatally diagnosed trisomy 21, 13 and 18 [37]. Termination of pregnancy may raise ethical and legal issues. In Austria, according to the Austrian Criminal Code (‘Strafgesetzbuch’) § 97, termination of pregnancy is not a criminal offence under certain conditions. This contains, among others, the first three months of pregnancy (‘Fristenlösung’), life-threatening risks or risks of serious harm for the physical or mental health of the pregnant woman that cannot be averted in any other way, or a serious risk that the child will be severely mentally or physically disabled. Hence, the maternal risk of pregnancy must also be considered, especially in cases of foetal diseases with an unfavourable prognosis. For example, pregnant women with foetal diandric triploidy have an increased risk for maternal complications, including pre-eclampsia [38, 39]. Interdisciplinary commissions can assist in difficult situations during the decision-making process as to whether termination can be offered at an institution. This could reduce the burden on individual decision-makers. The goal should be early diagnosis and decision-making without putting pressure on the expectant parents.

### Intrauterine therapies for specific genetic disorders

Prenatal detection of genetic disorders may be relevant in individual cases, as this could allow special intrauterine or immediate perinatal drug therapy. Current international guidelines, for instance, recommend prenatal testing for achondroplasia to permit early postnatal initiation of new therapies [40]. The advantages of prenatal initiation of specific therapies could be a more tolerogenic immune system and different pharmacodynamics, e.g. due to a more permissive blood-brain barrier. The initiation of specific therapies before the onset of irreversible disease sequelae is also mentioned [41]. X-linked hypohidrotic ectodermal dysplasia due to genetic deficiency of ectodysplasin A (EDA) is one example in this context. Prenatal intraamniotic application of a recombinant EDA1 replacement protein and subsequent oral intake by the foetus at the time of sweat gland development enabled gland development and pilocarpine-inducible sweating in all six treated patients with a follow-up up to six years. These patients also had more permanent teeth. In contrast, three patients who received the drug shortly after birth showed no sweat glands and no sweating ability at the age of 12–60 months [42, 43]. Tuberous sclerosis is a disease with autosomal dominant inheritance. Thereby, pathogenic loss-of-function disease-causing variants in the tuberous sclerosis complex (TSC) 1 or 2 gene result in hyperactivation of the mammalian target of rapamycin (mTOR) pathway [44]. It can be associated with cardiac rhabdomyomas, leading to significant cardiac strain and arrhythmia in some patients. Individual case reports show regression of foetal rhabdomyomas and improvement in cardiac function with oral maternal-foetal therapy using the mTOR inhibitor sirolimus [45–47]. However, undesirable side effects such as foetal growth restriction, maternal hypertriglyceridemia and maternal cough have been reported [46–48]. The latter led to discontinuation of therapy after five weeks in one case, whereby the newborn died of a therapy-resistant tachycardia [48]. Oral maternal administration of 5 mg risdiplam daily in the third trimester until delivery, followed by postnatal therapy, has been described in one case of confirmed spinal muscular atrophy (SMA) [49]. The splice modifier risdiplam has so far been used postnatally to increase and maintain functional survival motor neuron protein levels [50]. Drug concentration at delivery was 33 % in amniotic fluid and 69 % in cord blood. No typical signs of SMA were declared during the follow-up period. The optic nerve and left midbrain hypoplasia, as well as the associated mild right hemiparesis and the global developmental delay, were classified as non-therapy related [49]. Others argue that the time of therapy initiation could have been too late to prevent developmental deficits [51]. The potential fetotoxic risk described in animal models should also be taken into account [52]. For children under two months of age, a dose of 0.15 mg/kg is specified in the prescribing information, which is below the level measured in umbilical cord blood. From two years of age and a body weight of at least 20 kg, 5 mg daily is recommended therein [50]. Cohen et al. [53] present another example of a successful intrauterine therapy in a pregnant woman with a history of three children suffering from early-onset Pompe’s disease. This lysosomal storage disease is characterised by an acid α-glucosidase enzyme deficiency and a risk of prenatal onset of hypertrophic cardiomyopathy. One of the previous pregnancies was terminated, and two children died at eight and 29 months, respectively. In the current pregnancy, intrauterine enzyme-replacement therapy with alglucosidase alfa was administered through the umbilical vein every two weeks between 25 and 35 weeks of gestation after cross-reactive immunologic material-negative infantile-onset Pompe’s disease was confirmed by invasive diagnostics. At 13 months and under standard postnatal therapy, the patient showed normal cardiac and motor function. Individual case reports describe prenatal meconium ileus resolutions in F508del homozygous foetuses through maternal triple therapy with the agents elexacaftor, tezacaftor and ivacaftor – known cystic fibrosis transmembrane conductance regulator modulators [54, 55].

Intrauterine transfusions for foetal anaemia in rare foetal haemoglobinopathies such as beta thalassaemia have also been described [56]. In addition, there are studies on in utero haematopoietic stem cell transplantations for alpha thalassaemia major (ClinicalTrials.gov Identifier: NCT02986698). Nipocalimab is a neonatal Fc receptor blocker, which is intended to reduce maternal and foetal immunoglobulin G (IgG) levels [57]. Two ongoing phase 3, randomised, multicentre studies are examining the use of nipocalimab in pregnancies at risk for foetal and neonatal alloimmune thrombocytopenia (ClinicalTrials.gov Identifier: NCT06449651) and in pregnancies at risk for severe haemolytic disease of the foetus and newborn (ClinicalTrials.gov Identifier: NCT05912517) when foetal antigens match predefined maternal antibodies [57, 58].

For some genetic diseases, specific treatments already exist for children but are so far not tested prenatally. Several preclinical studies investigate the possibility of implementing gene therapies in utero [59]. At the same time, it is important to consider potential foetal and maternal side effects and toxicities and reduce maternal and foetal risks.

## Conclusion

Prenatal diagnostic tests for genetic abnormalities in the foetus have now become part of routine antenatal care and can be applied with low procedure-related risks. However, the ongoing development of increasingly complex genetic analyses and the interpretation of the results pose a major challenge. Providing adequate care for families with foetal genetic aberrations therefore requires an interdisciplinary team of obstetricians, prenatal specialists, geneticists, psychologists, midwives, neonatologists and paediatric specialists. New prenatal treatment options for specific genetic disorders appear promising. Intrauterine therapeutic approaches are expected to become increasingly important in the future. Though, many of these therapies are currently limited to rare diseases and small numbers of cases. Larger prospective clinical studies are necessary to evaluate therapy efficacy and safety.
